# Green-synthesized ZnO nanoparticles for efficient atrazine detection: electrochemical and computational investigations

**DOI:** 10.1039/d5na00595g

**Published:** 2025-09-18

**Authors:** Simranjeet Singh, Pavithra N., Radhika Varshney, Ashutosh Panchal, Nabila Shehata, Nadeem A. Khan, Joginder Singh, Praveen C. Ramamurthy

**Affiliations:** a Interdisciplinary Centre for Water Research (ICWaR), Indian Institute of Science Bengaluru Karnataka 560012 India praveen@iisc.ac.in onegroupb203@gmail.com; b Department of Materials Engineering, Indian Institute of Science Bengaluru Karnataka 560012 India; c Environmental Science and Industrial Development Department, Faculty of Postgraduate Studies for Advanced Sciences, Beni-Suef University Egypt; d Civil Engineering Department, College of Engineering, King Khalid University Abha 61421 Saudi Arabia; e Department of Botany, Nagaland University HQRS: Lumami 798627 Nagaland India

## Abstract

Developing sustainable and efficient methods for detecting environmental contaminants like atrazine (ATZ) is critical for environmental monitoring. This study uses a green approach to synthesise zinc oxide nanoparticles (g-ZnO NPs), employing an aqueous extract of *Haldina cordifolia* leaves as a natural reducing and stabilizing agent. The synthesized g-ZnO NPs were characterized using techniques such as XRD, TGA, SEM, UV-Vis spectroscopy, XPS, and FTIR to confirm their crystalline structure, morphology, optical properties, and functional groups. These nanoparticles demonstrated excellent sensitivity for the detection of ATZ, a widely used herbicide, *via* an electrochemical approach. The molecular docking simulations also predicted a favourable affinity of ZnO towards ATZ *via* hydrogen bonding. The sensor developed exhibited high selectivity for ATZ detection, achieving an LLOD of 0.41 μg mL^−1^ within a linear range of 0.5 to 3 μM. Its practicality was validated in different types of water where the recovery rates ranged from 87.26% to 94.8% in STP water and from 90.52% to 95.66% in DI water, highlighting their reliability in real-world applications. In this study, *Haldina cordifolia* is being explored for the first time to synthesize g-ZnO NPs, which are then utilized for the electrochemical detection of ATZ. The biosynthetic approach not only provides an eco-friendly route for nanoparticle synthesis but also enhances the potential for rapid and reliable detection of ATZ in water and STP samples.

## Abstract 2

Detection and docking studies of ATZ utilizing a green approach.

## Graphical abstract

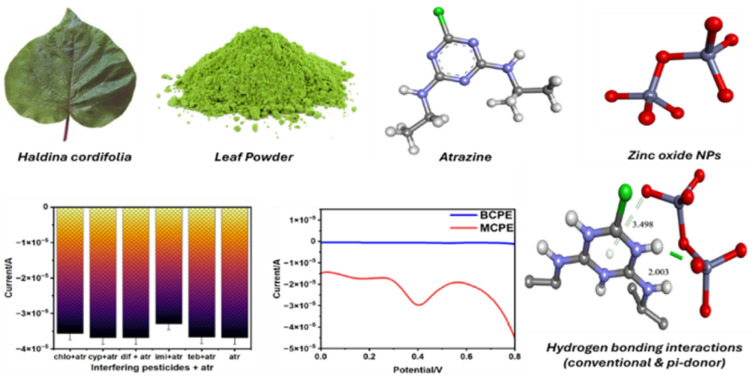

## 1. Introduction

In modern agriculture, substantial quantities of diverse toxic chemicals are employed to increase productivity and protect crops from various pests.^1^ These agents, primarily organic compounds, including pesticides, demonstrate significant environmental persistence^2^ along with a propensity for bioaccumulation and harmful effects on non-target species.^3^ For instance, organochlorinated pesticides represent a category of pesticides that are characterized by their significant chemical stability and resistance to degradation.^4^ These characteristics enhance their environmental longevity and capacity for bioaccumulation within the food chain, hence posing dangers to ecosystems and human health.^5^ Although prohibited or severely limited in most nations, residues of organochlorinated pesticides continue to be identified in soils, water, and live creatures owing to their persistent environmental presence.^6^ As a result, there is an increasing apprehension about the elevated health risks associated with human exposure to these environmental pollutants.^7^ Consequently, the surveillance of these pesticides has emerged as a crucial goal in contemporary analytical chemistry.

Atrazine (ATZ), a prevalent herbicide, is mostly utilized to manage grasses and broadleaf weeds in crops such as maize and sugarcane.^8^ Being water-soluble, ATZ is prone to leaching and runoff, which results in the contamination of surface and groundwater.^9^ Its half-life ranges from 20 to 100 days in soil and may reach 330–385 days in other systems, depending on environmental conditions.^10^ In small quantities, ATZ is classified as a type C carcinogen and an endocrine disruptor, affecting the hormonal system.^11^ Recognized as a priority pollutant, its use in agriculture has been prohibited under Slovak legislation (Parliamentary Act No. 364/2004 Coll. and Government Ordinance No. 296/2005 Coll.) and European Commission legislation (Commission Decision 2004/248/EC).^12,13^ In most parts of the world, ATZ is listed among hazardous substances and is a focus of initiatives aimed at reducing water pollution caused by emerging contaminants.^14^ These concerns underscore the growing demand for advanced, sensitive, and selective analytical techniques to monitor residual levels of banned pesticides in the environment.

Recent literature highlights numerous reports detailing analytical methods for the detection of ATZ and other triazine herbicides. Among these, spectrophotometric techniques and chromatographic methods, particularly high-performance liquid chromatography (HPLC) and gas chromatography (GC), have emerged as the most prevalent approaches.^8^ These chromatographic techniques are favoured for their exceptional sensitivity and selectivity in detecting trace levels of triazine herbicides. However, their application is often constrained by the need for complex sample preparation, typically involving pre-concentration steps prior to analysis. Additionally, these methods are associated with high costs, significant time investment, solvent waste generation, and the requirement for highly skilled operators, which can limit their feasibility for routine analytical use.^15^

Electrochemical methods are particularly well-suited for the environmental monitoring of ATZ due to their simplicity, cost-effectiveness, and adequate sensitivity.^12,15,16^ These methods offer a viable alternative to conventional spectrophotometric and chromatographic techniques. For the detection of ATZ, various materials have been reported in the literature, such as MnO_2_–NiO^17^ and Co_3_O_4_–C/Fe-MOF.^18^ Moreover, materials such as UiO-67,^19^ reduced-graphene oxide,^20^ ZnFe_2_O_4_,^21^ activated carbon,^22^ and N–NiO@N–Fe_3_O_4_@N–ZnO^23^ have been utilized for the adsorption or degradation of ATZ. However, most of these materials are not environmentally friendly and pose significant toxicity, such as the use of Ni, which is a well-known carcinogen.^24^ Consequently, the development of novel, simple, and environmentally friendly electrochemical tools for the sensitive detection of ATZ residues is of critical importance for sustainable environmental monitoring. The usage of green synthesized nanomaterials^25,26^ and nanocomposites^27^ offers the advantages of being environmentally friendly as it uses less hazardous precursors and is cost-effective.

*Haldina cordifolia* (syn. *Adina cordifolia*) is a large deciduous tree of the Rubiaceae family, commonly known as Yellow Teak, Kadami, Haldu, or Saffron Teak. Known for its anti-cancer, anti-tuberculosis,^28^ anti-inflammatory,^29^ anti-microbial,^30^ anti-ulcer properties,^31^ and drug discovery research,^32^ this plant is also capable of acting as a reducing and capping agent for the green synthesis of various materials, owing to its rich phytochemical composition that indicates the presence of flavonoids, saponins, alkaloids, and terpenes.^28^ In the context of sensor development, the introduction of these bio-functional groups (such as flavonoids and alkaloids) can improve analyte interaction, electron transfer kinetics, and overall sensitivity.

Therefore, *Haldina cordifolia* is being explored for the first time to synthesize green ZnO nanoparticles (ZnO NPs), which are then utilized for the electrochemical detection of ATZ. The synthesized ZnO, known for its biocompatibility, high surface area, and excellent electron transfer properties, significantly enhanced the sensor's sensitivity and selectivity in detecting ATZ. This study underscores the efficacy of incorporating green materials such as ZnO in the realm of electrochemical ATZ detection. It encapsulates the conceptualization, fabrication, and evaluation of a green sensor designed to precisely detect and quantify ATZ levels.

## 2. Materials and methods

### 2.1 Chemicals

All chemicals, media, and solvents utilized in this study were of analytical reagent (AR) grade and were sourced from various vendors in Bangalore, Karnataka, India. Zinc acetate, ethanol, atrazine, difenoconazole (Dif), cypermethrin (Cyp), chlorimuron-ethyl (Chlo), imidacloprid (Imi), tebuconazole (Teb), and other chemicals were obtained from Sigma-Aldrich.

### 2.2 Biogenic synthesis

The synthesis was performed following our previous protocol with slight modifications.^33^*Haldina cordifolia* leaves were washed with Milli-Q water to remove contaminants and then dried for one week at 35° ± 2 °C under dark conditions. Approximately 2 g of leaves were ground, dissolved in 100 mL Milli-Q water, and heated at 75° ± 2 °C for 20 min. The solution was filtered, yielding a brown leaf extract that was stored at 4 °C. A 0.01 M zinc acetate dihydrate solution was prepared in deionized water. To synthesize green ZnO NPs, 100 mL of zinc acetate solution was mixed with 5 mL of extract and incubated at 90° ± 2 °C with shaking at 200 rpm for 60 min. A light brown precipitate formed, which was centrifuged at 8000 rpm for 8 min after three washes with water to remove contaminants and was stored.

### 2.3 Molecular docking simulations

Molecular docking simulations were performed to predict the possibility of interactions in the ATZ–ZnO system. The structure of ZnO was downloaded from the Materials Project (mp-2133)^34^ in the CIF format, loaded to Vesta software,^35^ and saved as a protein data bank file (PDB). The structure of ATZ was downloaded from PubChem (CID: 2256)^36^ in the SDF format of the 3D structure available. Both the structures were auto-optimised in Avogadro^37,38^ using default parameters and then saved as a PDB file. The ATZ was assigned as the macromolecule, and ZnO was the ligand for the molecular docking studies. Blind docking was performed using AutoDock Tools (ADT),^39^ wherein a grid box of 40 × 40 × 40 points with 0.375 spacing was used for running AutoGrid4, and a genetic algorithm with 500 runs and a population size of 300 was utilised as a search parameter for running AutoDock4. The topmost conformation ranked by energy obtained after docking was saved and analysed using ADT and Discovery Studio visualizer (DSV).^40^

### 2.4 Electrochemical detection of ATZ

Differential Pulse Voltammetry (DPV) and Cyclic Voltammetry (CV) analyses were conducted using a CHI660D electrochemical workstation procured from CH Instruments Inc., Austin, TX, USA. The experiments were carried out in a three-electrode system configuration, comprising a saturated calomel electrode (SCE) as the reference electrode, a carbon paste electrode (CPE) as the working electrode, and a platinum wire as the counter electrode. The bare carbon paste electrode (BCPE) was fabricated by mixing 0.243 g of fine graphite powder with 40 μL of silicone oil to produce a homogeneous paste. This paste was applied to the electrode loop and polished to achieve a smooth and uniform surface. The BCPE was electrically connected to the CHI660D system using a copper wire. For the preparation of the modified carbon paste electrode (MCPE), 6 mg of synthesized ZnO NP composite was thoroughly mixed with 0.24 g of graphite powder and 40 μL of silicone oil to form a uniform paste. The MCPE was fabricated using the same procedure as the BCPE. Repeatability was assessed by independently fabricating a MCPE using the same preparation procedure. The electrochemical response of each electrode was measured in freshly prepared buffer containing ATZ, under consistent experimental conditions. Electrochemical measurements for both electrodes were conducted in a 0.1 μM Tris–HCl buffer solution. For real-time analysis, water samples were collected and filtered to remove suspended solids. Samples were spiked with ATZ to simulate real-world contamination. Electrochemical measurements were carried out using the MCPE, and the standard addition method was applied to account for matrix effects. Recoveries were calculated to assess sensor performance in complex water matrices. All electrochemical measurements were performed in triplicate.

## 3. Results and discussion

### 3.1 Docking results

**Fig. 1:**
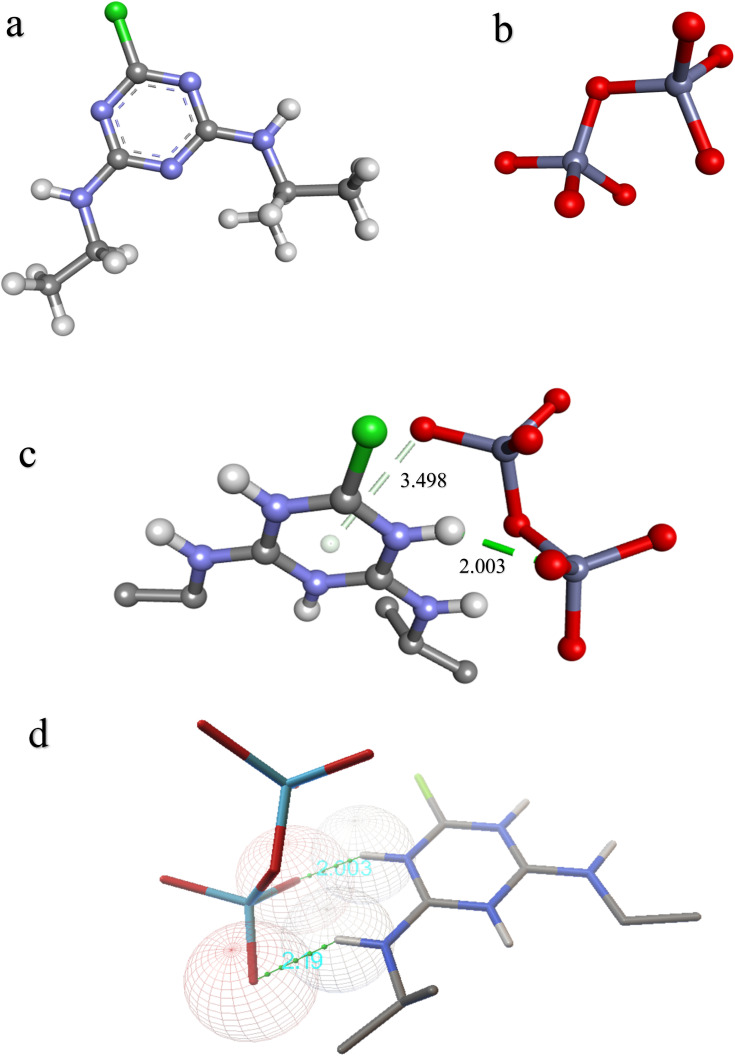
Molecular structures of (a) ATZ and (b) ZnO, and topmost docked conformation as represented in (c) DSV and (d) ADT.

The topmost docked conformation obtained in molecular docking simulations had a binding energy of −2.04 kcal mol^−1^, with an estimated inhibition constant (dissociation constant of macromolecule-ligand docked complex) of 32.14 mM (at 298.15 K). The negative binding energy obtained suggests a favourable affinity of ZnO towards ATZ. Furthermore, the type of interaction as predicted using DSV is hydrogen bonding (conventional and pi–donor). Similar predictions were also observed using the ADT (2 conventional hydrogen bonds) (Fig. 1). The difference in the nature of the hydrogen bonds predicted could be due to the different parameters of both software. Nevertheless, the hydrogen bonding interactions are suggested in both cases for the ZnO–ATZ system.

### 3.2 Phase purity characterization of g-ZnO NPs

**Fig. 2:**
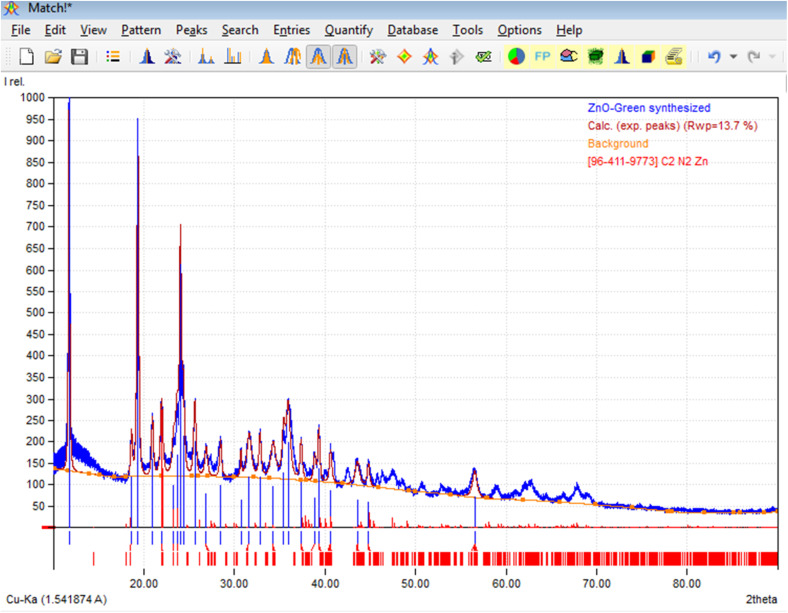
XRD pattern of g-ZnO developed from HC leaves.

To determine the purity and composition of the g-ZnO NPs developed from *Haldina cordifolia* (HC) leaves, the XRD spectrum of g-ZnO NPs was investigated (Fig. 2). All characteristic peaks noticed for g-ZnO NPs are in good agreement with those taken from the COD (Crystallography Open Database) card no. 96-411-9773.^41^ According to the indexing pattern, it can be confirmed that the XRD pattern of g-ZnO NPs corresponds to an orthorhombic unit cell with *a* = 1.22842 nm, *b* = 0.76537 nm and *c* = 0.75151 nm.

### 3.3 Spectroscopic analysis of plant material and g-ZnO NPs

**Fig. 3:**
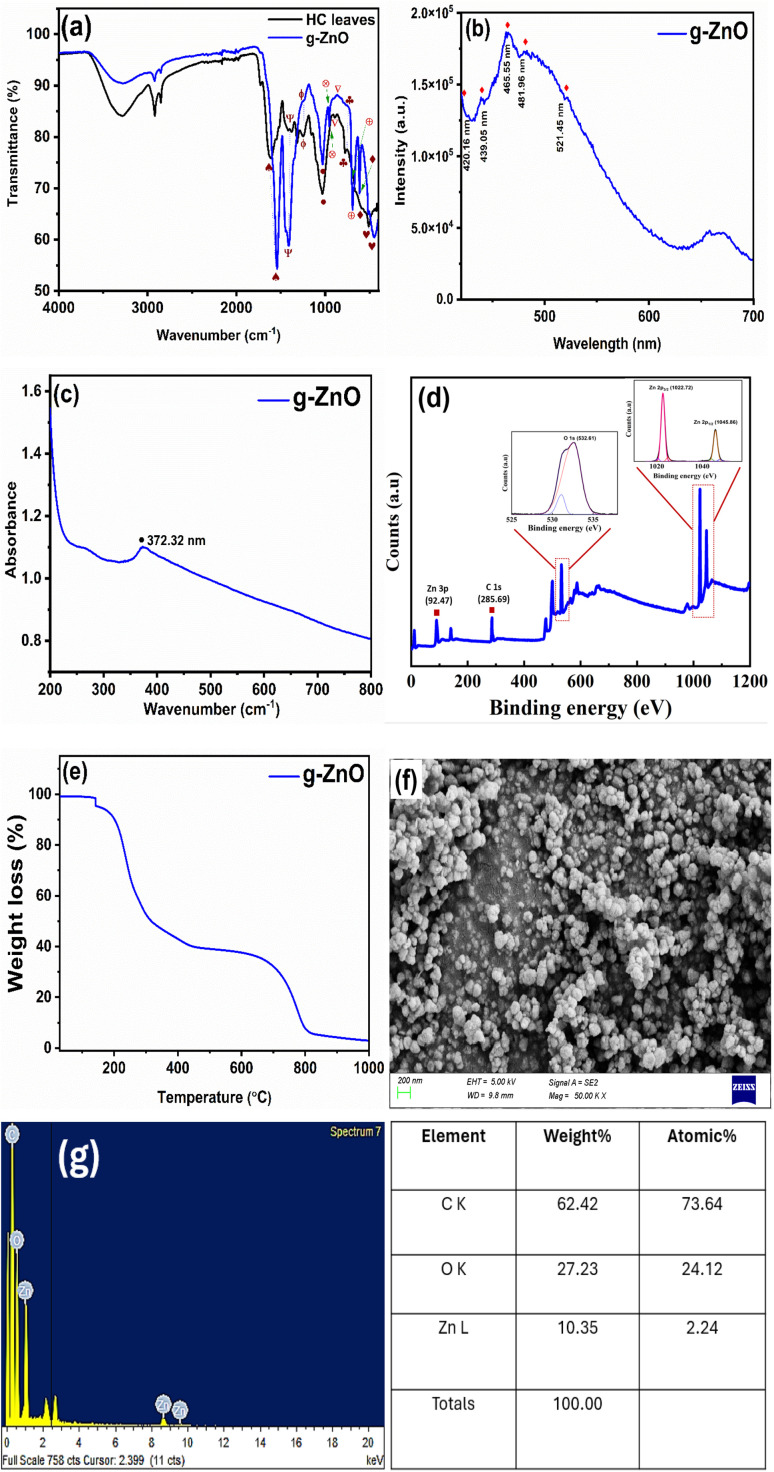
Characterization of the developed g-ZnO NPs: (a) FTIR, (b) PL, (c) UV, (d) XPS (inset shows high-resolution spectra for the Zn 2p and O 1s regions of ZnO as well as C 1s), (e) TGA, (f) SEM image and (g) EDX.

The infrared transmission spectra of HC leaves and the developed g-ZnO NPs were recorded in the range of 4000–400 cm^−1^ (Fig. 3a). The band at 3276.65 cm^−1^ in the spectrum of HC leaves corresponds to the O–H group of water and plant extract components^42^ and –NH or –OH- stretching in amino acids, alcohols and phenols.^43^ This band shifted from 3276.65 cm^−1^ in the spectrum of HC leaves to 3268.71 cm^−1^ in the spectrum of ZnO. The stretching vibration of C–H^43^ located at 2917.83 cm^−1^ in HC leaves shifted to 2919.87 cm^−1^ in the ZnO spectrum. The stretching vibrations of C=O and C–H^44^ appeared at 2850.24 cm^−1^ in both spectra. The unconjugated stretching vibration of C=O located at 1739.74 cm^−1^ in the spectrum of HC leaves shifted to 1725.67 cm^−1^ in the ZnO spectrum. A peak at 1336.62 cm^−1^ in the HC spectrum, which corresponds to the presence of O–H in-plane deformation,^44^ is absent in the ZnO spectrum. The C–O of the guaiacyl ring^44^ at 1250.34 cm^−1^ in the HC leaves shifted to 1236.39 cm^−1^ in the developed ZnO. Additionally, C–O of primary alcohols and C–H deformation in guaiacyl^44^ which was recorded at 1034.47 cm^−1^ in HC leaves shifted to 1027.86 cm^−1^ in the ZnO spectrum. It is worth mentioning that the intensity of transmittance of all above peaks increased in the ZnO spectrum compared to HC leaves, corresponding to the involvement of these functional groups in the development of g-ZnO NPs. On the other hand, the transmittance % of some peaks in the developed ZnO decreased compared to that of HC leaves such as C=O and C=N^42,43,45^ and the stretching of C–H and C–N of aromatic amines^42^ combined with a shift from 1613.89 cm^−1^ and 1375.76 cm^−1^ in HC leaves to 1544.82 cm^−1^ and 1411.21 cm^−1^ in the ZnO spectrum, respectively. The lower intensities of both peaks suggest a reduction in the proportions of these functional groups in the developed ZnO compared to HC leaves. The peak corresponding to C–H out-of-plane^44^ and glucose ring stretch at 894.12 cm^−1^ (ref. 46) in the spectra of HC disappeared in the ZnO spectrum. Some new peaks appeared in the ZnO spectrum such as the stretching vibration of N–H^46^ (949.93 cm^−1^) and aromatic rings (615.01 cm^−1^ and 692.20 cm^−1^).^45^ Finally, the peak at 440.21 cm^−1^ in the spectrum of the developed ZnO corresponds to a distinctive stretching of ZnO.^46^ The presence of different functional groups such as alcohols, phenols aldehydes, ketones, and carboxylic acid suggests that the bio-entities may play a significant role in the development and stabilization of ZnO NPs, which is in agreement with previous studies.^42,43,47^

### 3.4 Light emission and optical properties of g-ZnO NPs

The PL spectrum for g-ZnO (excitation = 300 nm) is depicted in Fig. 3b. The spectrum shows some clear bands around 420.16, 465.55, 481.96, 657.67, and 670.76 nm and two weak peaks at 439.05 and 521.45 nm. The band at 420.15 nm is attributed to the band edge emission and near band edge emission of ZnO.^48^ Blue emission at 439.05 nm comes from the donor level of interstitial Zn to the acceptor level of Zn vacancies.^49,50^ The blue–green emission band at 465.55 nm is developed by an electron radiative transition from shallow donor levels, developed by O vacancies, to the valence band.^51,52^ Also, previous work suggested that the band at 465.55 nm is attributed to Zn vacancies.^53^ A common band at 550 nm in the PL spectrum of ZnO, which corresponds to the deep level transitions in ZnO,^48^ appears here as a weak peak at 521.45 nm.^48^ The green emission band at 481.96 nm is attributed to transitions involving O vacancies.^54^ There is an unknown band around 670 nm that may correspond to some minor impurities arising from the functional group of the HC leaves. Fig. 3c shows the absorbance spectrum of ZnO in the range from 200 to 700 nm. A typical exciton absorption at 372.32 nm is recorded in the absorption spectrum of g-ZnO NPs at room temperature, which agrees with previous studies.^55–57^

### 3.5 Surface morphology, chemistry and thermal stability analysis

The XPS spectrum of the g-ZnO sample (Fig. 3d) confirms the presence of Zn and O, in addition to C as a reference. The high-resolution spectrum of Zn 2p shows that the binding energies of Zn 2p_1/2_ and Zn 2p_3/2_ are 1045.86 and 1022.72 eV, respectively. However, the difference between these two values is the spin–orbit splitting (SOS). The SOS here is 23.13 eV, which is equal to that of ZnO.^45,58^ The peak at 532.61 eV in the O spectrum can be attributed to O 1s in the lattice of ZnO. Fig. 3e shows the TGA measurement results of g-ZnO NPs. As can be seen from Fig. 3e, the weight loss of ZnO occurs in two main steps. The first step is in the range of 142 °C to about 434 °C, demonstrating evaporation of surface water. The second weight loss occurred in the region from 624 °C to 820 °C, which was attributed to the combustion of organic species in the sample. Fig. 3f and g show the morphology and EDX of g-ZnO NPs. The structure is irregularly close to a spherical-like structure. The nanoparticles appear stacked on clusters with uniform particle size (<200 nm). The variation in size may be attributed to the synthesis conditions, which include the type and concentration of precursor and reaction time. According to Subramaniam *et al.*, the size distribution of the developed g-ZnO can be controlled by fine-tuning these parameters.^59^ The EDX spectrum shows the optical absorption peaks of g-ZnO NPs, which are attributed to the surface plasmon resonance of g-ZnO NPs, and the table (on the right) confirms the presence of Zn (10.35%) and O (27.2%). The high % of carbon in the sample may be attributed to the action of X-ray emissions on the degradation of polysaccharides, amino acids, sugars and proteins verifying the hydrocarbon composition developed in the medium,^60^ in addition to the adsorption of atmospheric carbon, which is a common cause for the high carbon percentage in the XPS analysis of green synthesized metal oxides.^61^

### 3.6 Electrochemical sensing of ATZ using ZnO NPs

#### 3.6.1 Electrochemical performance of CP/CPE

**Fig. 4:**
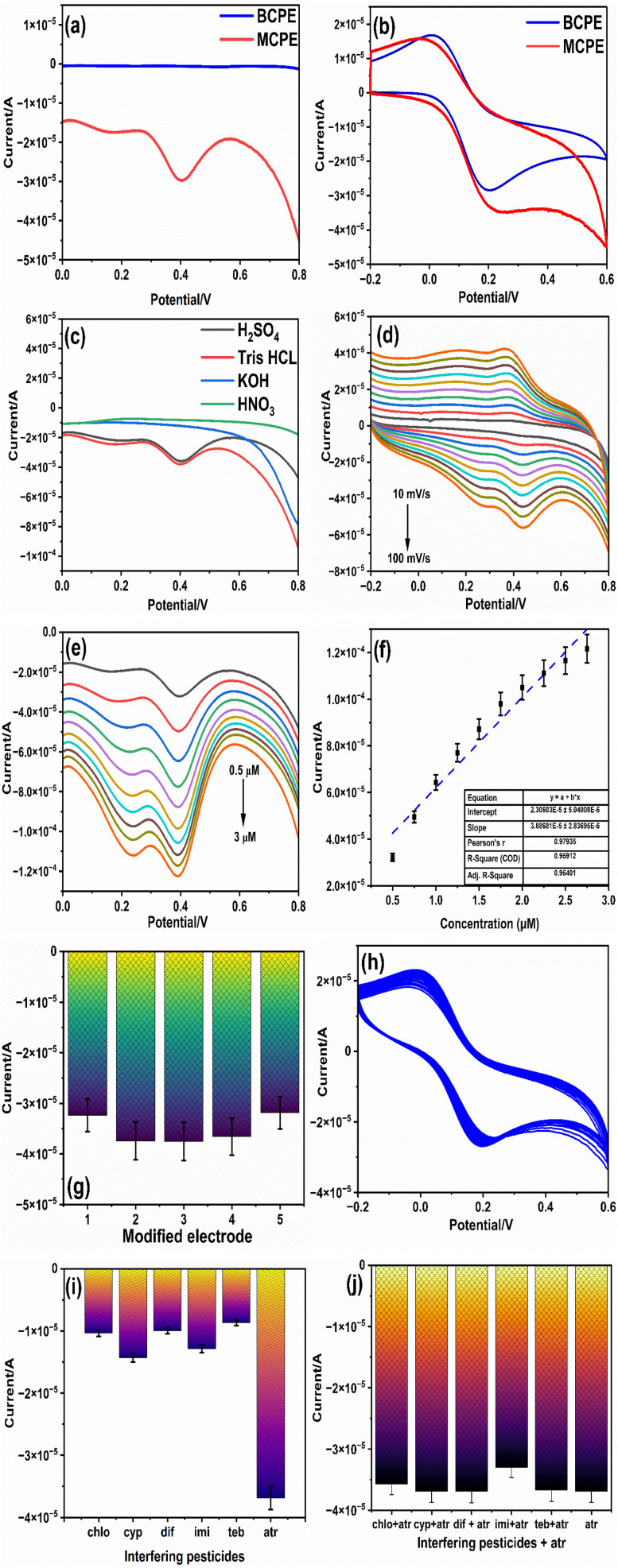
(a) Effect of bare and modified electrodes on ATZ detection, (b) electrochemical characterization of ATZ in ferricyanide solution, (c) effect of buffer on ATZ oxidation, (d) effect of the scan rate, (e) effect of concentration variation of ATZ on the modified ZnO NP electrode, (f) line plot of current intensity *versus* the concentration of ATZ, (g) repeatability of ZnO NPs/CPE, (h) stability of ZnO NPs/CPE over 50 cycles, (i) effect of interfering molecules, and (j) effect of interfering molecules along with ATZ on ZnO NPs/CPE.

The electrocatalytic performance of the ZnO NP-modified carbon paste electrode (ZnO NPs/CPE) for ATZ detection was systematically evaluated in 0.1 M Tris–HCl buffer, which served as the supporting electrolyte/buffer. Differential Pulse Voltammetry (DPV) was utilized as the analytical technique to investigate the electrocatalytic behavior of ZnO NPs towards ATZ within the potential range of 0.0 V to 0.8 V. This technique was chosen for its high sensitivity and ability to distinguish the oxidation peak of ATZ. Fig. 4a depicts the comparative voltammograms of unmodified and ZnO NP-modified electrodes in the presence of 10 μM ATZ solution. The results clearly demonstrate a substantial enhancement in the oxidation current of ATZ when using the modified electrode. Specifically, the oxidation peak current increased by approximately three-fold at a peak potential of 0.4 V. The absolute areas of the BCPE and MCPE were estimated from Fig. 4b and found to be 1.1 × 10^−5^ and 1.57 × 10^−5^, respectively. This marked improvement is attributed to the exceptional electrocatalytic properties of the ZnO NPs, which enhance electron transfer kinetics and provide a larger active surface area for the electrochemical catalyses to occur. The significant increase in the oxidation current not only underscores the effectiveness of ZnO NPs in catalyzing the electrochemical oxidation of ATZ but also highlights the potential of the modified electrode for sensitive and selective detection of ATZ. Consequently, the ZnO NPs/CPE system was deemed highly efficient and was selected for further studies to evaluate its electrocatalytic efficiency and practical applicability in ATZ detection. This enhanced performance establishes the ZnO NP-modified electrode as a promising platform for environmental monitoring and pesticide analysis.

#### 3.6.2 Effect of buffer

In electrochemical studies, the selection of an appropriate electrolyte is vital for accurately assessing electron charge transport and ensuring reliable measurements. Diffusion, governed by concentration gradients, facilitates the movement of analytes from regions of higher to lower concentration within the electrochemical cell. Convection effects, which can interfere with analyte transport, are minimized by maintaining the system under static conditions. Migration, where charged species are influenced by electric fields, is effectively suppressed by employing highly concentrated electrolytes. The type and concentration of the electrolyte play a pivotal role in determining the extent to which the electrolyte reaches the electrode surface to maintain balance in the electron charges, thereby influencing electron transfer kinetics. To identify the optimal electrolyte for ATZ detection, the response of the ZnO NP-modified carbon paste electrode (ZnO NPs/CPE) was evaluated using four different electrolytes: 0.1 M H_2_SO_4_, 0.1 M KOH, 0.1 M HNO_3_, and 0.1 M Tris HCl buffer. 10 μM atrazine was analysed over a potential range of 0.0 V to 0.8 V. The results are illustrated in Fig. 4c, indicating that 0.1 M Tris–HCl buffer produced the highest peak response compared to the other supporting electrolytes. The definite peaks were observed at 0.4 V and 0.2 V in the presence of ATZ, demonstrating superior sensitivity and signal clarity in the Tris–HCl buffer.

Based on these observations, 0.1 M Tris–HCl buffer was selected as the suitable electrolyte for further experiments which provided a stable environment for efficient electron transfer and its enhanced current response in the presence of ATZ involving the ZnO NP-modified electrode. This choice of the electrolyte contributes to the improved sensitivity and reliability of the electrochemical detection system, assisting its application in environmental monitoring.

#### 3.6.3 Effect of the scan rate

To evaluate the effect of the scan rate on the voltammetric behavior of ATZ at the ZnO NPs/CPE electrode, with the aim of understanding the kinetics of the electrode reactions involving ATZ a study was conducted. Fig. 4d illustrates the cyclic voltammograms of the ZnO NPs/CPE system with scan rates ranging from 10 to 100 mV s^−1^ and in the presence of 20 μM ATZ. The results show that an increase in the scan rate leads to a proportional increase in the redox peak currents of ATZ. A slight deviation in the peak potentials toward more positive values is also observed at higher scan rates, indicating an enhancement in the electron transfer kinetics.

##### 3.6.3.1 Electrode kinetics and electron transfer mechanism using Laviron analysis

In electrochemical studies, both anodic peak current (*I*_pa_) and cathodic peak current (*I*_pc_) at different scan rates show a linear relationship with the scan rate (*I*_p_ ∝ *ν*^1/2^), indicating that a surface-based/diffusion process governs the electrochemical responses.

The electron transfer coefficient (*α*) and the number of electrons (*n*) in the redox process of ATZ were estimated using Laviron's equation, which describes the peak potential dependent on the scan rate (*ν*) in a quasi-reversible electrochemical system. According to Laviron, the anodic and cathodic peak potentials are given by the equations*E*_pa_ = *E*^0^ + (2.303*RT*/(1 − *α*)*nF*)log *ν**E*_pc_ = *E*^0^ − (2.303*RT*/*αnF*)log *ν*

The Laviron equations of ATZ anodic and cathodic currents are estimated to be *E*_pc_ = (6.2 × 10^−5^ log *ν*) − 8.6 × 10^−5^ and *E*_pa_ = 1.01 × 10^−4^ − (7.2 × 10^−5^ log *ν*). From these equations, the slopes corresponding to the cathodic and anodic processes are found to be 6.2 × 10^−5^ and 7.2 × 10^−5^ V, respectively, which were used to calculate the electron transfer coefficient (*α*) of 0.537 and the number of electrons (*n*) to be 2.

This behaviour underscores the role of electrode kinetics in influencing redox activity. These results illustrate the significance of scan rate optimization in the study of redox processes and offer important insights into the electrochemical kinetics of ATZ at the ZnO NPs/CPE electrode. The results demonstrate the electrode's effectiveness in facilitating electron transfer, making it a promising platform for the electrochemical analysis of ATZ.

#### 3.6.4 Effect of concentration variation

To estimate the electrocatalytic performance of the ZnO NP-modified electrode, the Differential Pulse Voltammetry (DPV) technique was employed to examine its response to various concentrations of ATZ, ranging from 0.5 μM to 3 μM, in 0.1 M Tris–HCl buffer. The DPV results, depicted in Fig. 4e, illustrate a significant increase in the peak oxidation current at 0.4 V as the ATZ concentration increases. This trend shows the modified electrode's enhanced capability to strengthen the electrochemical signal in response to higher analyte concentrations, emphasizing its efficacy as a sensing platform. A regression plot, shown in Fig. 4f, was plotted from the peak current data obtained during the DPV current response obtained for ATZ concentration. The plot exhibits a linear relationship between the peak current (*I*_cp_) and ATZ concentration, described by the equation *I*_cp_ = (3.886 × 10^−5^)Conc + (2.3 × 10^−5^), with a correlation coefficient *R*^2^ value of 0.95. This linearity underscores the precision and reliability of the electrode's response over the tested concentration range, establishing it as a dependable tool for quantitative analysis. To assess the sensitivity of the modified electrode, the limit of detection (LOD) was calculated using the equation LOD = 3SD/slope, where SD denotes the standard deviation of the blank measurements. The calculated LOD was found to be 0.41 μg mL^−1^, demonstrating the electrode's capability to detect low concentrations of ATZ with high sensitivity. This low detection limit highlights the modified electrode's potential for trace-level analysis, which is critical for environmental applications.

Overall, the results illustrate that the ZnO NP-modified electrode offers a sensitive and reliable tool for the electrochemical detection of ATZ. Its linearity, low detection limit, and ability to produce a peak response to increasing ATZ concentration underscore its applicability for environmental monitoring and pesticide analysis as tabulated in Table 1.

**Table 1:** Comparative analysis of electrodes for ATZ detection

Modified electrode	Linear range	LOD	Reference
SiO_2_@ATZ-MIP nanoparticles	—	1.8	62
Ha006a/MCPE	10–100	5.4	63
Tyrosinase immobilization on polyvinyl alcohol with styryl-pyridinium groups	10–100	1.3	64
Molecularly imprinted conducting polymer	0.001–15 000	0.4	65
GNPs	0.21–2.1	0.016	66
Boron-doped diamond electrode	0.05–40	0.01	67
Cell-free sensor	—	20	68
MIP-based screen-printed potentiometric cell	0.5–5	0.4	69
Electrospun SnO_2_ nanofiber	1 × 10^−9^–1	9 × 10^−7^	70
ZnO NPs	0.5–3	1.9	Present work

#### 3.6.5 Stability and repeatability

The effectiveness of the ZnO NP-modified carbon paste electrode (ZnO NPs/CPE) as a sensor was measured through experiments estimating its repeatability and stability. To examine repeatability, five parallel ZnO NPs/CPEs were separately fabricated using the same preparation method. Each electrode was tested for its electrochemical response to 10 μM ATZ in freshly prepared buffer solutions. The results, shown in Fig. 4g, reveal a relative standard deviation of 6.7% in the peak current, indicating excellent reproducibility of the sensor fabrication process and measurement reliability.

The stability of ZnO NPs/CPE was further investigated using cyclic voltammetry (CV) over 50 consecutive cycles within a potential range of −0.2 V to 0.6 V at a scan rate of 50 mV s^−1^. The results demonstrated only an ∼8.9% reduction in the oxidation peak current of ATZ in the 50th cycle compared to the first cycle, highlighting the electrode's strong electrochemical stability under repeated use, as shown in Fig. 4h. These findings confirm that the ZnO NPs/CPE sensor is both reliably reproducible and stable, making it a robust platform for the electrochemical detection of ATZ. The sensor's low RSD and minimal degradation over multiple cycles underscore its potential for consistent and long-term applications in environmental monitoring and analytical sensing.

#### 3.6.6 Effect of interfering molecules

A critical feature of a high-performance sensor is its ability to selectively detect a specific analyte in the presence of other interfering substances that may also undergo oxidation within the same potential range. To evaluate the selectivity of the ZnO NPs/CPE sensor, experiments were conducted using the DPV technique by using 20 μM concentrations of various potential interferents, namely difenoconazole (Dif), cypermethrin (Cyp), chlorimuron-ethyl (Chl), imidacloprid (Imi), and tebuconazole (Teb), in 0.1 M Tris–HCl buffer. Fig. 4i shows that the peak current for ATZ remained relatively high when compared to other interferents. This highlights the inherent selectivity of the sensor towards ATZ amid potential interferences. Further, to assess the performance of ZnO NPs/MCPE in a real-time analysis, equal concentrations of ATZ and various interfering molecules were introduced into the reaction cell in a 0.1 M Tris–HCl buffer. The results, depicted in Fig. 4j, showed a decrease in the sensitivity of the modified electrode when interference was added. Despite this decrease, the effect of interference ions at the same concentration was minimal on the detection of ATZ. This demonstrates that ZnO NPs/CPE maintains a high degree of selectivity for ATZ even in the presence of interference from other substances.

In summary, the ZnO NPs/CPE sensor exhibits robust selectivity for detecting ATZ, making it well-suited for applications where interference from other chemicals is a concern. This selectivity ensures reliable and accurate detection of ATZ, enhancing the sensor's performance in complex analytical environments.

#### 3.6.7 Real-time detection of ATZ in spiked and wastewater samples

The ZnO NPs/CPE sensor was tested to detect ATZ in wastewater and distilled water. Wastewater samples were collected from a treatment plant and centrifuged at 8000 rpm for 10 minutes to eliminate suspended particles. The supernatant of wastewater was then spiked with various concentrations of ATZ. Peak current measurements were taken, and ATZ levels were quantified using a linear calibration equation. Table 2 shows the sensor's performance in different samples. The recovery rate was highest in distilled water compared to wastewater, likely due to the presence of interfering molecules. Despite this, the sensor showed reliable performance in wastewater, demonstrating its potential for real-world applications.

**Table 2:** Recovery percentage of the sensor using real-time samples

Samples	Added concentration (μM)	Obtained concentration (*n* − 3)	Recovery percentage (%)
STP water	6	5.69 ± 2.13	94.8%
	12	10.46 ± 0.61	87.26%
	18	16.79 ± 2.18	92.12%
Distilled water	6	5.74 ± 0.86	95.66%
	12	10.863 ± 1.64	90.52%
	18	16.98 ± 0.31	94.33%

## 4. Conclusion

This study successfully demonstrates the synthesis of g-ZnO NPs using a green and eco-friendly approach, leveraging the natural reducing and stabilizing properties of *Haldina cordifolia* leaf extract. Comprehensive characterization confirmed the crystalline structure, morphology, optical properties, and functional groups of the synthesized g-ZnO NPs. The molecular docking simulations conducted predicted the favorable interactions between ZnO and ATZ. The nanoparticles exhibited remarkable sensitivity and specificity for ATZ detection through an electrochemical approach, with a linear detection range of 0.5 μM to 3 μM and a lower detection limit of 0.41 μg mL^−1^. Additionally, the recovery rates of 87% to 95.66% in DI water and STP water samples highlight their reliability in real-world applications. This biosynthetic method not only provides a sustainable route for nanoparticle production but also establishes an efficient, cost-effective, and environmentally friendly platform for monitoring ATZ contamination in DI water and wastewater samples. These findings open avenues for further research into the green synthesis of nanomaterials for environmental and agricultural applications.

## Conflicts of interest

There are no conflicts to declare.

## Data Availability

The datasets generated and/or analyzed during the current study are available from the corresponding author upon reasonable request. All relevant experimental data, including electrochemical measurements, characterization results, and molecular docking outputs, have been thoroughly documented and can be provided to support the findings of this research.
